# Response to the Letter regarding “Sugammadex vs neostigmine in post-anesthesia recovery: A systematic review and meta-analysis”

**DOI:** 10.17305/bb.2025.13781

**Published:** 2025-12-24

**Authors:** Ni Zhu, Yongli Li

**Affiliations:** 1 Department of Anesthesiology, Hospital of Chengdu University of Traditional Chinese Medicine, Sichuan, China

**Keywords:** Heterogeneity, hypnotic depth, airway safety, statistical methods

## Abstract

This response addresses feedback on our systematic review and meta-analysis comparing sugammadex with neostigmine for neuromuscular block reversal. We acknowledge high heterogeneity for time-based outcomes, likely due to differences in clinical settings and anesthetic/surgical protocols, but pooled effects consistently favored sugammadex for faster and more complete reversal. We agree hypnotic depth and other perioperative factors may modify emergence and airway safety, yet these variables were inconsistently reported and could not be analyzed quantitatively. We also clarify that time outcomes were synthesized using standardized mean differences to account for different reporting units, and any presentation inconsistencies will be corrected. Overall, our findings support pharmacologic superiority of sugammadex with reductions in selected complications, while emphasizing that broader recovery quality may not uniformly improve and should be interpreted in clinical context.

Dear Editor,

We appreciate the reader’s insightful comments on our systematic review and meta-analysis. We welcome the opportunity to clarify our work and engage in constructive scientific dialogue. Below is our point-by-point response.

**1. Heterogeneity:** We acknowledge the high statistical heterogeneity (*I*^2^ > 90%) observed for certain outcomes, particularly time-based measures such as recovery to train-of-four (TOF) ≥ 0.9 and extubation time [1]. This heterogeneity likely reflects variability in clinical settings, anesthetic regimens, and surgical types across the randomized controlled trials included in our analysis—a common challenge in meta-analyses of pragmatic clinical outcomes [2]. Despite this variability, the direction and statistical significance of the pooled effect consistently favored sugammadex, underscoring its pharmacologic efficacy. We agree that caution is warranted in interpreting pooled estimates under conditions of high heterogeneity and emphasize in our discussion that individual clinical contexts should guide application.

**2. Hypnotic depth and airway safety:** The reader presents a pertinent concern about the impact of hypnotic depth, specifically the minimum alveolar concentration (MAC) at reversal, on the quality of emergence and airway safety [3]. Our analysis primarily focused on comparing the reversal efficacy and postoperative complications between the two pharmacologic antagonists, as stated in our objective. While factors such as volatile anesthetic concentration, patient age, and surgical duration are important modifiers of recovery physiology, they were not consistently reported in the included studies and, therefore, could not be incorporated into a quantitative subgroup analysis. We concur that these are significant areas for future research to refine clinical guidance.

**3. Effect size reporting:** We apologize for any confusion regarding the presentation of effect sizes. In our results, time-to-event outcomes (e.g., recovery to TOF ≥ 0.9, extubation time) were analyzed using the standardized mean difference (SMD) to integrate studies that reported time on different scales (e.g., seconds vs minutes). The numerical values in the tables and figures correspond to SMDs; any inconsistency was unintentional and will be rectified in future versions. The large magnitude of the SMD for recovery to TOF ≥ 0.9 (–3.45) reflects the consistent and significant reduction in time with sugammadex across studies.

**4. Statistical methods and contextualization:** We employed conventional random-effects meta-analysis, which is a standard and appropriate approach for synthesizing randomized controlled trials with expected clinical diversity. We acknowledge that advanced methods (e.g., trial sequential analysis) can provide additional insights [4]. Our conclusion presents a nuanced perspective, indicating that while sugammadex effectively reduces certain postoperative complications—such as postoperative nausea and vomiting (PONV), postoperative pulmonary complications (PPCs), and bradycardia—this expedited reversal does not result in measurable enhancements in overall recovery quality. This aligns with the reader’s observation that pharmacologic superiority does not always translate into uniform clinical benefit across all patient-centered outcomes.

## Conclusion

We thank the reader for highlighting these important methodological and interpretive considerations. Our review provides robust evidence that sugammadex facilitates faster and more complete pharmacologic reversal of neuromuscular blockade and reduces several specific postoperative complications compared to neostigmine. We fully agree that anesthesiologists must integrate this pharmacologic advantage within a broader clinical context that includes hypnotic depth, patient factors, and comprehensive recovery protocols.
